# Antitussive effects of the peripherally restricted GABA_B_ receptor agonist lesogaberan in guinea pigs: comparison to baclofen and other GABA_B_ receptor-selective agonists

**DOI:** 10.1186/1745-9974-8-7

**Published:** 2012-10-01

**Authors:** Brendan J Canning, Nanako Mori, Anders Lehmann

**Affiliations:** 1 Johns Hopkins Asthma and Allergy Center, 5501 Hopkins Bayview Circle, Baltimore, Maryland, 21224, USA; 2 AstraZeneca R&D Mölndal, SE-431 83, Mölndal, Sweden

**Keywords:** Gastroesophageal reflux, Esophagus, LES relaxation, C-fiber, TRPV1, Lesogaberan

## Abstract

**Background:**

Gastroesophageal reflux disease (GERD) is a common cause of chronic cough. Both acid and nonacid reflux is thought to play a role in the initiation of coughing and cough hypersensitivity. The GABA_B_ receptor agonist lesogaberan was developed as a peripherally restricted anti-reflux therapy that reduces the frequency of transient lower esophageal sphincter relaxations (TLESR; the major cause of reflux) in animals and in patients with GERD. GABA_B_ receptor agonists have also been shown to possess antitussive effects in patients and in animals independent of their effects on TLESR, suggesting that lesogaberan may be a promising treatment for chronic cough.

**Methods:**

We have assessed the direct antitussive effects of lesogaberan (AZD3355). The effects of other GABA_B_ receptor agonists were also determined. Coughing was evoked in awake guinea pigs using aerosol challenges with citric acid.

**Results:**

Lesogaberan dose-dependently inhibited citric acid evoked coughing in guinea pigs. Comparable effects of the GABA_B_ receptor agonists baclofen and 3-aminopropylphosphinic acid (3-APPiA) on cough were also observed. Baclofen produced obvious signs of sedation and respiratory depression. By contrast, both lesogaberan and 3-APPiA (both inactivated centrally by GABA transporters) were devoid of sedative effects and did not alter respiratory rate.

**Conclusions:**

Together, the data suggest that lesogaberan and related GABA_B_ receptor agonists may hold promise as safe and effective antitussive agents largely devoid of CNS side effects.

## 

Cough is one of the most commonly reported symptoms amongst patients seeking medical advice. Acute cough is triggered primarily by viral infections, while the most common causes of chronic cough are asthma, upper airway inflammatory disorders, and gastroesophageal reflux disease (GERD). Therapeutics used specifically for the treatment of cough are either minimally effective or have unwanted side effects that limit their utility. In patients with chronic cough, treatment of their underlying disease can improve patient quality of life and reduce coughing. But for many patients with chronic, troublesome cough, even after aggressive medical treatment of their underlying illnesses, cough can remain a significant health problem that adversely impacts quality of life. New and more effective and selective treatments for cough thus represent an unmet need in respiratory medicine [1,2].

Agonists of the metabotropic GABA_B_ receptor such as baclofen have been evaluated for their utility in targeting a number of peripheral disorders thought to involve aberrant reflexes and sensations including pain, overactive bladder, hiccups, tetanus/spasticity, and headache [3-10]. GABA_B_ receptor agonists have also been evaluated for their effects on airways hyperresponsiveness, GERD and cough [11-20]. Although clinical benefit has been reported in these latter studies, a sedative effect of baclofen has also been noted [21,22]. Ideally, an effective treatment for cough would prevent cough through direct effects on sensory nerves innervating the airways and independent of any significant CNS-dependent side effects. A therapy that targets both cough and GERD would be especially desirable, given the association between these conditions.

Key to the side effect profile of systemically administered GABA_B_ receptor agonists is their CNS penetrance and susceptibility to inactivation by uptake [23,24]. GABA and analogs of GABA are subject to uptake in the central nervous system by the 4 identified GABA transporters (GAT1-GAT4). Baclofen is not a substrate for uptake and can act centrally when administered peripherally [25]. By contrast, 3-APPiA is a GABA_B_ receptor agonist that is inactivated centrally by transport [24-26]. There are reports of antitussive effects of 3-APPiA in guinea pigs and cats [27,28]. It is thus possible that GABA_B_ receptor agonists work peripherally to prevent vagal reflexes.

Lesogaberan (AZD3355) is a GABA_B_ receptor agonist with limited CNS side effects that was developed for the treatment of GERD [20,25,29-31]. Like 3-APPiA, lesogaberan is inactivated centrally by GAT-dependent transport [25]. The purpose of this study was to evaluate the effects of lesogaberan and other GABA_B_ receptor agonists on citric acid induced coughing in guinea pigs.

## Methods

The institutional animal care and use committee approved all of the studies described below. Male Hartley guinea pigs (200-400 g, Charles River) were placed in a flow through chamber filled with air by an air pump. A pressure transducer was connected to the outflow of the chamber to monitor respiratory efforts and coughing in response to citric acid challenge. Data was recorded digitally using a Biopac data acquisition system.

Guinea pigs were pretreated 30 minutes prior to citric acid challenge with vehicle, lesogaberan (0.3-10 mg/ kg), baclofen (0.3 and 3 mg/ kg), 3-APPiA (0.3 and 3 mg/ kg) or SKF97541 (0.1 and 0.3 mg/ kg), administered by subcutaneous injection. After a 10 minute equilibration period in the exposure chamber when basal respiratory rate was monitored, guinea pigs were then challenged with increasing concentrations of citric acid (0.01, 0.1, 0.3 and 1 M), delivered by nebulizer (particle size: <5 μm) connected in series with the air pump. Each dose was delivered for 5 minutes, with a 5 minute interval in between doses. The total number of coughs evoked by each concentration of citric acid both during the 5 minute challenge and during the 5 minutes following the challenge was determined. The results of these studies are presented as the cumulative number of coughs evoked.

**Figure 1:**
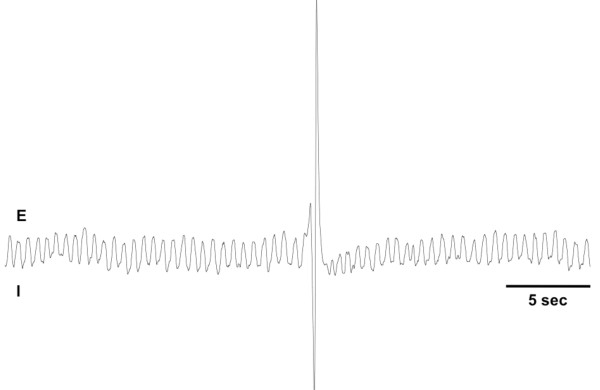
**A representative trace of coughing evoked by a citric acid challenge to an awake guinea pig is depicted.** Inspiratory (I) efforts produce a negative pressure in the chamber, with expiratory (E) efforts producing positive pressures. These traces were used to measure respiratory rate at the outset of each experiment (breaths/ min), the time to first cough following initiation of the citric acid challenges, the Peak to Peak (P-P) pressures associated with cough (measured by comparing the P-P pressures associated with coughing, expressed as a percentage of the P-P pressures measured at eupnea), the total number of coughs evoked by each dose of citric acid and the total number of coughs evoked cumulatively by all doses of citric acid studied.

Cough was defined visually and by the characteristic pressure changes in the chamber, reflecting an enhanced inspiratory effort followed by a forceful expiratory effort, with expiratory pressure changes ≥ 500% of the pressures associated with expiration during eupnea, all occurring in less than 1 second. Coughing is readily differentiated from enhanced breaths (sighs), often associated with stimuli inducing bronchospasm, which have a roughly symmetric pressure signature (with equally enhanced inspiratory and expiratory pressures) and slower cycle (Figure 1). In these healthy, young animals and with the cough evoked by citric acid aerosols, the tussive responses evoked were presumed and indeed likely to be the result of a direct effect of the citric acid on airway sensory nerves. By extension, any antitussive effects of the compounds studied were likely to be the result of a direct effect on cough, and not the consequence of any effects on underlying pathology (e.g. GERD).

The results are presented as a mean ± sem of n experiments, where n is the number of guinea pigs studied. Guinea pigs were challenged only once (to each dose of citric acid) in this unpaired experimental design. Differences amongst group means were evaluated by 1 way analysis of variance. A p-value of less than 0.05 was considered significant.

### Reagents

Baclofen and citric acid were purchased from Sigma (St. Louis, MO). AstraZeneca (Mölndal, Sweden) provided lesogaberan, 3-APPiA and SKF97541. All reagents were dissolved in 0.9% NaCl solution.

## Results

**Figure 2:**
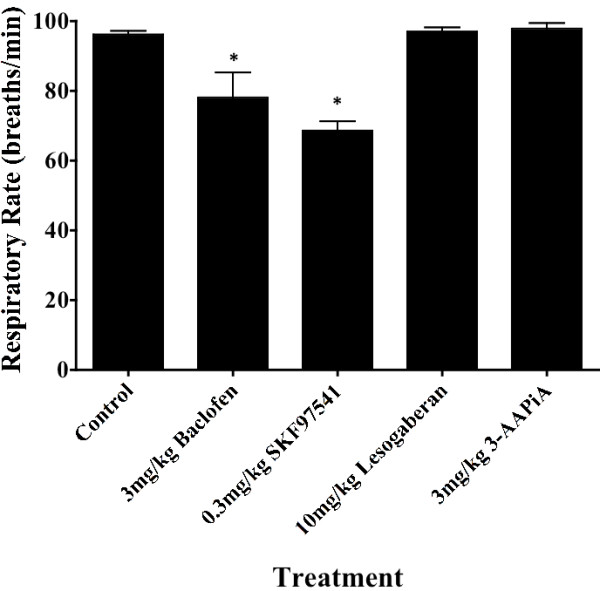
**GABA**_**B**_**receptor agonists have differential effects on respiratory rate in awake guinea pigs. Each bar represents the mean ± sem of 5–15 experiments.** An asterisk (*) indicates that the treatment reduced respiratory rate relative to that measured in animals pretreated with vehicle (p < 0.05).

Baseline respiratory rate in control animals averaged 96 ± 1 breaths/ min (n = 15). At a dose of 3 mg/ kg, both baclofen and SKF97541 reduced baseline respiratory rate (78 ± 7 and 69 ± 3 breaths/ min, respectively; n = 5-6; p < 0.05) and produced obvious signs of sedation and lethargy. By contrast, 3 mg/ kg 3-APPiA and lesogaberan, even up to a dose of 10 mg/ kg, were without effect on respiratory rate (98 ± 2 and 97 ± 1 breaths/min, respectively; n = 7-8; p > 0.1) and produced no other overt signs of sedation or lethargy (Figure 2).

Citric acid inhalation evoked concentration-dependent coughing in the awake guinea pigs. No animals in any of the treatment groups coughed in response to the citric acid vehicle (water) inhalation, while 56/61 animals coughed at least once to 0.3 M citric acid challenge. The highest concentration of citric acid was not always well-tolerated. Five-minute challenges with 1 M citric acid were interrupted in 2/15 control experiments due to labored breathing. All but 3 of 41 animals pretreated with baclofen, 3-APPiA or lesogaberan completed the 1 M citric acid challenges. The coughing evoked by citric acid had a characteristic pattern of single, powerful coughs, occasionally 2 on consecutive breaths, but never the paroxysmal coughing we reported previously in studies of bradykinin evoked coughing [32,33]. No signs of tachyphylaxis were apparent in these studies. Thus, if a robust cough response was evoked by a lower concentration of citric acid, subsequent challenge with a higher dose of citric acid still evoked coughing.

**Figure 3:**
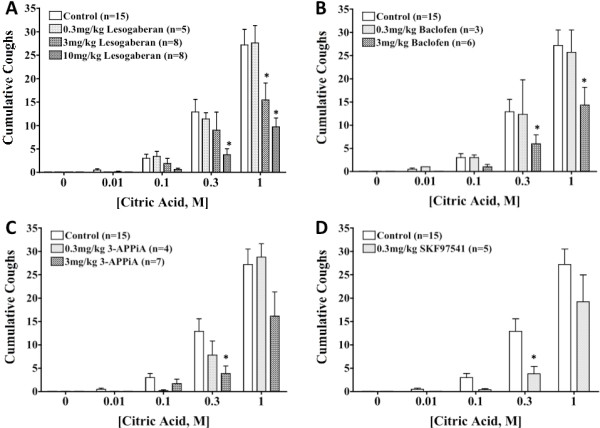
**Effects of GABA**_**B **_**receptor agonists on citric acid evoked coughing. Each bar represents the mean ± sem of 3–15 experiments.** An asterisk (*) indicates a significant reduction in the number of cumulative coughs relative to vehicle control (p < 0.05).

Baclofen, 3-APPiA and lesogaberan all inhibited citric acid induced coughing (Figure 3). The effects of all of these drugs were found to be dose-dependent. Lesogaberan was equipotent to baclofen in these studies (but, as mentioned above, without coincident sedative effects and respiratory depression). The sedating effects of SKF97541 were so profound that its antitussive actions were not extensively studied. Baclofen, 3-APPiA and lesogaberan all slightly reduced the percentage of animals coughing to any given dose of citric acid, and reduced the number of coughs evoked by given doses of citric acid. None of the drugs studied had any pronounced effects on the time to onset of coughing, nor on the peak pressures produced during the cough (Table 1).

**Table 1:** **Effects of GABA**_**B **_**agonists on the percentage of animals coughing, time to onset of coughing and on peak expiratory pressures during cough**

				**%Animals Coughing to Citric Acid**	
**Treatment**	**n**	**Time to 1**^**st**^**Cough (min)**	**-P Pressures (%control)**	**0.01M**	**0.1M**
Vehicle control	15	2.0±03	1144±102%	27% (4/ 15)	93% (14/ 15)
0.3 mg/ kg baclofen	3	2.4±0.6	837±187%	100% (3/ 3)	100% (3/ 3)
3 mg/ kg baclofen	6	1.4±0.3	705±110%	0% (0/ 6)*	50% (3/ 6)
3 mg/ kg SKF97541	5	2.7±1.0	852±92%	0% (0/ 5)*	60% (3/ 5)
0.3 mg/ kg 3-APPiA	4	2.8±0.7	762±121%	0% (0/ 5)*	80% (4/ 5)
3 mg/ kg 3-APPiA	7	3.3±0.7	986±120%	0% (0/ 7)*	57% (4/ 7)
1 mg/ kg lesogaberan	5	1.7±0.2	1426±142%	0% (0/ 5)*	80% (4/ 5)
3 mg/ kg lesogaberan	8	2.1±0.6	1100±144%	13% (1/ 8)	63% (5/ 8)
10 mg/ kg lesogaberan	8	3.0±0.6	855±99%	0% (0/ 8)*	50% (4/ 8)

The data are presented as the %animals coughing or as the mean ± sem of n experiments where n is a single animal studied in a non paired experimental design. The time to first cough was measured during challenges with 0.3 M citric acid. The peak to peak (P-P) pressures associated with cough were measured for the first 5 coughs evoked during challenge, regardless of citric acid concentration. An asterisk (*) indicates a statistically significant difference from that observed in vehicle treated animals (p < 0.05).

## Discussion

Lesogaberan is a GABA_B_ receptor agonist with a limited CNS side effect profile. This compound has been evaluated as a treatment for GERD, a primary cause of chronic cough [20,25,29-31]. In the present study, lesogaberan was found to be an effective antitussive agent, preventing citric acid evoked coughing in conscious guinea pigs in a dose-dependent manner but without coincident sedative effects or respiratory depression. We found this compound was as potent and effective as baclofen, a GABA_B_ receptor agonist used clinically for the treatment of several disorders. Unlike lesogaberan, however, the antitussive effects of baclofen were accompanied by undesirable side effects, including sedation, respiratory depression and a trend towards a decrease in peak cough pressures. Based on these results, we conclude that lesogaberan may hold promise for the treatment of acute and chronic cough and may have a better side effect profile than baclofen.

The four GABA_B_ receptor agonists used in this study are essentially identical in receptor pharmacological profile. All are selective GABA_B_ receptor agonists, with SKF97541, 3-APPiA and lesogaberan sharing structural similarities. Key to their differential side effect profile, however, is their susceptibility to uptake by GABA transporters [23-25]. Baclofen and SKF97541 are not transported, whereas both 3-APPiA and lesogaberan are substrates for GAT. Thus, the site of action for the latter 2 compounds is thought to be limited to peripheral locations and perhaps central locations with limited GAT expression [19,25]. The marked respiratory depression and lethargy induced by SKF97541 and baclofen but not by lesogaberan and 3-APPiA we observed are consistent with the known susceptibility to uptake of these compounds.

Our results confirm and extend the studies by Bolser et al. [27,28,34], who reported that both baclofen and 3-APPiA prevented coughing in guinea pigs and cats. Because 3-APPiA was more effective administered peripherally than centrally, the authors concluded that GABA_B_ receptor agonists prevent cough at least in part through peripheral sites of action. But a central site of action of these drugs cannot be entirely dismissed. We found that intraperitoneally administered baclofen inhibits cough [33], but we also reported that baclofen microinjected into the nTS inhibits cough in anesthetized guinea pigs [35]. Bolser et al. concluded that baclofen works primarily via central effects [28,34]. Similarly, Callaway and King [36] found that baclofen inhalation prevented citric acid induced alterations in respiratory pattern (possibly by inhibiting bronchospasm) but was without effect on citric acid induced cough. Perhaps cell groupings in the brainstem, accessible to peripherally administered agonists and on the fringe of the blood brain barrier, are targeted by these drugs. For a central site of action to be viable, agonists such as 3-APPiA and lesogaberan must penetrate brainstem regions relevant to cough and attain sufficient concentrations to prevent or blunt synaptic transmission. There is conflicting evidence relating to blood-borne access and GABA uptake mechanisms in nTS [37-43].

GABA_B_ receptor activation might prevent coughing through peripheral inhibitory effects on bronchopulmonary vagal afferent nerves. The vagal afferent nerves regulating cough in guinea pigs are C-fibers arising from the jugular ganglia, and cough receptors, terminating in the larynx, trachea and mainstem bronchi and with cell bodies in the nodose ganglia [32,44]. Both of these vagal afferent nerve subtypes are responsive to acid but largely insensitive to changes in airway luminal pressure, stretch or airway smooth muscle contraction. The cough receptors are, however, insensitive to capsaicin. Given that Bolser et al. studied the antitussive effects of baclofen and 3-APPiA with capsaicin as the tussive stimulus, the available data would suggest that if GABA_B_ receptor agonists are working peripherally, they act at least in part through effects on airway vagal C-fiber terminals [27,28]. In fact, there is functional evidence to suggest that the vagal C-fibers regulating cough express GABA_B_ receptors on their peripheral terminals [45,46], but as yet, there is no evidence to suggest their activation would prevent action potential discharge. Interestingly, however, there are several studies showing direct inhibitory effects of GABA_B_ receptor agonists on the excitability of vagal afferent nerves, including those innervating the stomach and esophagus [47-50].

Non-neuronal sites of action for GABA_B_ receptor agonists are also possible. Acid evoked cough may be mast cell dependent, and mast cell activation may trigger or at least modulate subsequently evoked cough [51,52]. GABA_B_ receptor agonists can attenuate allergen-induced inflammation, perhaps through effects on mast cell activation [53,54]. GABA_B_ receptors on non-neural airway cells has also been documented, including airway smooth muscle and epithelium [55,56], and we have described a transduction pathway dependent upon activation of chemosensory epithelial cells [57]. Endogenous GABA in the lung may also regulate cough [58]. Finally, GABA_B_ receptor agonists have also been shown to attenuate parasympathetic-cholinergic responses in the airways, which might also indirectly attenuate cough responses [59-61]. In general, however, these non-neural influences on cough would seem more likely to be modulatory than essential to evoked cough. Bronchospasm is not an effective stimulus for cough, and bronchodilators have modest, variable effects on evoked cough [44].

GABA_B_ receptor agonists may hold promise in the treatment of GERD due to their ability to prevent TLESR, the primary physiologic process responsible for reflux [15,17,18,62,63]. Compounds such as lesogaberan may offer a distinct advantage over conventional therapeutic approaches to GERD inasmuch as proton pump inhibitors and histamine H2 receptor antagonists do not prevent reflux, but rather, reduce the acid content of gastric fluid. While acid is thought to be the major trigger of symptoms and pathology in GERD, other components of refluxate such as pepsin likely contribute to the pathophysiology of this disease [64,65]. GERD is also a major cause of chronic cough [1,2]. Indeed, cough may be the only presenting symptom of GERD in some patients. The ability of compounds such as lesogaberan to prevent coughing evoked directly from the airways is another potential therapeutic advantage of GABA_B_ receptor agonists in the treatment of GERD. These attributes along with the limited side effect profile of lesogaberan relative to that of baclofen provides impetus for continued study of this compound as a treatment for both GERD and chronic cough.

## Competing interests

AL is an employee of AstraZeneca.

## Authors’ contributions

BC designed and conducted many of the experiments, interpreted the results and wrote multiple sections of the manuscript. NM conducted many of the experiments, analyzed the data and created the graphic summaries of the results. AL designed the experiments, interpreted the results and wrote multiple sections of the manuscript. All authors read and approved the final manuscript.

## Funding

This study was funded by AstraZeneca and by a grant from the National Institutes of Health (HL083192).
